# Acid Ceramidase Depletion Impairs Neuronal Survival and Induces Morphological Defects in Neurites Associated with Altered Gene Transcription and Sphingolipid Content

**DOI:** 10.3390/ijms21051607

**Published:** 2020-02-26

**Authors:** Kalia Kyriakou, Carsten W. Lederer, Marina Kleanthous, Anthi Drousiotou, Anna Malekkou

**Affiliations:** 1 Cyprus School of Molecular Medicine, P.O. Box 23462, 1683 Nicosia, Cyprus; kaliak@cing.ac.cy (K.K.); lederer@cing.ac.cy (C.W.L.); marinakl@cing.ac.cy (M.K.); anthidr@cing.ac.cy (A.D.); 2 Biochemical Genetics Department, The Cyprus Institute of Neurology and Genetics, P.O. Box 23462, 1683 Nicosia, Cyprus; 3 Molecular Genetics Thalassaemia Department, The Cyprus Institute of Neurology and Genetics, P.O. Box 23462, 1683 Nicosia, Cyprus

**Keywords:** acid ceramidase, SH-SY5Y, neurites, ceramide, lipidomics, Rho GTPases, LAMP1

## Abstract

The *ASAH1* gene encodes acid ceramidase (AC), an enzyme that is implicated in the metabolism of ceramide (Cer). Mutations in the *ASAH1* gene cause two different disorders, Farber disease (FD), a rare lysosomal storage disorder, and a rare form of spinal muscular atrophy combined with progressive myoclonic epilepsy (SMA-PME). In the absence of human in vitro neuronal disease models and to gain mechanistic insights into pathological effects of *ASAH1* deficiency, we established and characterized a stable *ASAH1* knockdown (*ASAH1*^KD^) SH-SY5Y cell line. *ASAH1*^KD^ cells displayed reduced proliferation due to elevated apoptosis and G1/S cell cycle arrest. Distribution of LAMP1-positive lysosomes towards the cell periphery and significantly shortened and less branched neurites upon differentiation, implicate AC for lysosome positioning and neuronal development, respectively. Lipidomic analysis revealed changes in the intracellular levels of distinct sphingolipid species, importantly without Cer accumulation, in line with altered gene transcription within the sphingolipid pathway. Additionally, the transcript levels for Rho GTPases (RhoA, Rac1, and Cdc42), which are key regulators of axonal orientation, neurite branching and lysosome positioning were found to be dysregulated. This study shows the critical role of AC in neurons and suggests how AC depletion leads to defects seen in neuropathology of SMA-PME and FD.

## 1. Introduction

Human acid ceramidase (AC) is an enzyme implicated in the metabolism of ceramide (Cer) and is encoded by the *ASAH1* gene. AC catalyzes the conversion of Cer into a fatty acid and sphingosine (Sph). AC is a lysosomal hydrolase with a pH optimum of 4.5–5.0, deficiency of which leads to the ultra-rare disorder known as Farber disease (FD) [1]. In most cases, FD is diagnosed early in life and characterized by subcutaneous nodules, deformed joints and progressive hoarseness [2]. Additionally, progressive neurologic deterioration was observed in some subtypes [2]. More recently, AC deficiency has been shown to cause a form of spinal muscular atrophy (SMA) associated with progressive myoclonic epilepsy (SMA-PME) [3] or SMA without PME [4], which are characterized by proximal muscle weakness and generalized atrophy of muscles due to degeneration of spinal motor neurons [4].

In mouse, complete knockout of AC leads to early embryonic lethality during the two- to four-cell stage transition, highlighting its critical role in embryonic development [5]. In zebrafish, morpholino knockdown of the *ASAH1* gene led to specific defects of branches of motor neurons, a phenomenon associated with increased apoptosis in the spinal cord in the absence of Cer accumulation, suggesting a functional role of AC in motor axon development and maintenance [3]. Additionally, AC was found to be elevated in Alzheimer’s disease brain, co-localizing with neurofibrillary tangles [6]. The neurological defects associated with AC deficiency have been studied in a knock-in mouse model, *Asah1*^P361R/P361R^, demonstrating abnormalities and accumulation of Cer and other sphingolipids in a broad array of neuronal and non-neuronal cell types and in the brain as a whole [7].

AC is considered a “rheostat” for maintaining the balance between the levels of the anti-proliferative Cer and the pro-apoptotic Sph and the mitogenic phosphorylated product sphingosine-1-phosphate (Sph-1P), which plays a role in protection against Cer-mediated apoptosis [8], in survival and in cellular proliferation [9]. Cer has a central role in sphingolipid metabolism, because it is converted to various metabolites such as sphingomyelin (SM), Sph, ceramide-1-phosphate (Cer-1P), and glucosylceramide (GluCer), and any imbalance in Cer levels may lead to significant changes in cell membranes, and affect processes such as apoptosis, signal transduction and gene expression [8,9].

The way AC affects the phenotype and the growth of neuronal cells is not currently understood. Several studies have shown that impairment of the Cer pathway can result in apoptosis of neuronal and oligodendroglial cells in neuroinflammatory and neurodegenerative disorders including Alzheimer’s disease, Parkinson’s disease, HIV-associated dementia, multiple sclerosis and amyotrophic lateral sclerosis [10]. Cer can play distinct roles in growth and differentiation of neuronal cells, depending on its concentration at different stages of neuronal growth, stimulus type and Cer species [11]. Particularly, Cer synthesis is essential to evoke axonal growth in hippocampal neurons [12,13] and dendritic growth in cerebellar Purkinje cells [14]. Whether, Cer induces differentiation or apoptosis also depends on the expression of specific neurotrophin receptors, which are again regulated by Cer concentration [15]. Studies regarding the influence of relatively simple sphingolipids such as Cer, GlcCer, and Shp-1P in the neuronal function have shown that sphingolipids regulate important aspects of neurons such as the neuronal differentiation, growth and survival. Moreover, the normal neuronal function requires the correct maintenance of an equilibrium of balance between specific sphingolipids [12,13,16,17,18].

In this study, we addressed the need for a human in vitro neuronal cell model for studying the effects of AC deficiency. We established and characterized a stable *ASAH1* knockdown model, based on the human neuroblastoma cell line, SH-SY5Y, which is well-characterized and widely used for the investigation of neurological disorders [19]. This study shows that the phenotypic defects in cell morphology of AC-depleted SH-SY5Y cells correspond to altered lipids and gene transcription within the sphingolipid pathway, and to altered transcription of the Rho GTPase family members. Our results connect the neuronal defects of AC depletion with the neurological pathology observed in SMA-PME and FD.

## 2. Results

### 2.1. Establishment of Stable ASAH1 Knockdown Cell Lines

**Figure 1:**
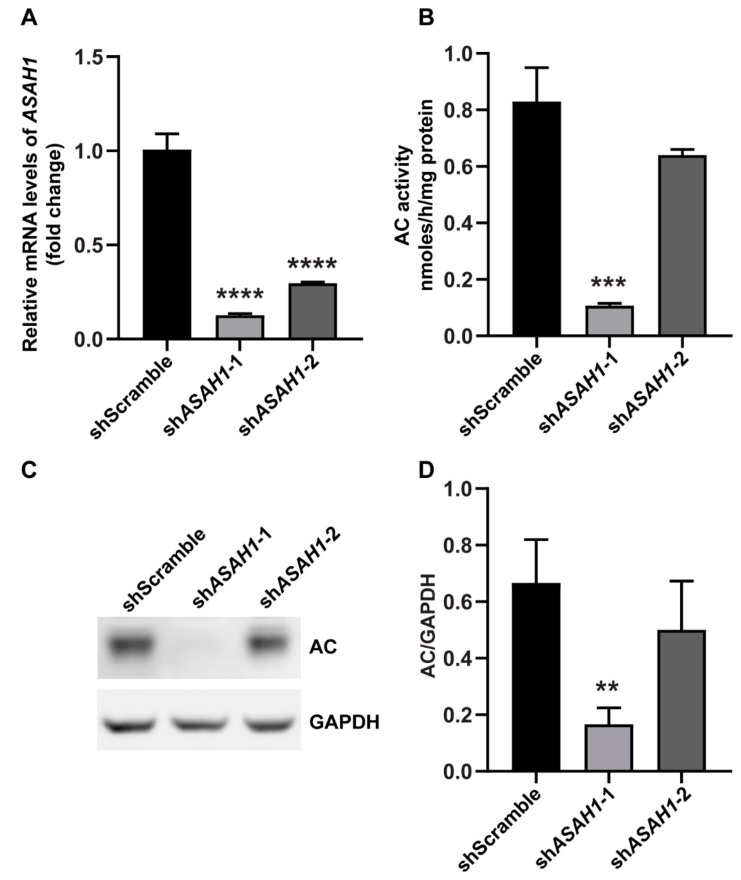
Knockdown of *ASAH1* by lentiviral shRNA transduction in SH-SY5Y cells. (**A**) Relative mRNA expression levels of *ASAH1* stably expressing either sh*ASAH1*-1 or sh*ASAH1*-2, compared to cells stably expressing a scrambled shRNA (shScramble), after normalization for endogenous *GAPDH* expression. Data are represented as the mean ± SEM of three independent triplicate experiments (one-way ANOVA analysis). (**B**) Enzyme activity of AC in sh*ASAH1*-1 and sh*ASAH1*-2 cells compared to shScramble cells. The activity was measured using an artificial fluorogenic substrate and was expressed as nmoles of product/h/mg protein. Data are expressed as the mean ± SEM of three independent triplicate experiments (one-way ANOVA analysis). (**C**) Representative immunoblot of AC in sh*ASAH1*-1, sh*ASAH1*-2 and shScramble cells. GAPDH was used as loading control. (**D**) Quantification of density of AC bands normalized by density of GAPDH bands for each sample detected by Western blot. Data are expressed as the mean ± SD of three independent experiments (one-way ANOVA analysis). ** *p* < 0.01, *** *p* < 0.001 and **** *p* < 0.0001 compared to shScramble.

We established two stable *ASAH1* knockdown cell lines of SH-SY5Y cells (sh*ASAH1*-1 and sh*ASAH1*-2) expressing two different shRNAs (shRNA1 and shRNA2) against different regions of *ASAH1* mRNA, using a lentiviral approach. A stable SH-SY5Y cell line expressing a scrambled shRNA sequence was also established to serve as control (shScramble). Reverse-transcription quantitative PCR (RT-qPCR) confirmed the efficient reduction of *ASAH1* mRNA in cells expressing the specific shRNAs of *ASAH1*, compared to shScramble cells. The mRNA expression level of sh*ASAH1*-1 was significantly decreased by 7.7-fold and sh*ASAH1*-2 was decreased by 3.4-fold compared to the shScramble (Figure 1A). Additionally, the relative enzymatic activity of AC in sh*ASAH1-1* and sh*ASAH1-2* cells was reduced to 0.11 nmoles/h/mg protein (10% of shScramble) and 0.64 nmoles/h/mg protein (60% of shScramble), respectively (Figure 1B). Likewise, immunoblotting showed 74% and 24% reduction of AC expression in sh*ASAH1-1* and sh*ASAH1-2* cells respectively, compared to shScramble cells (Figure 1C,D).

Combined, these results showed that *ASAH1* knockdown was more efficient for shRNA1 than for shRNA2.

**Figure 2:**
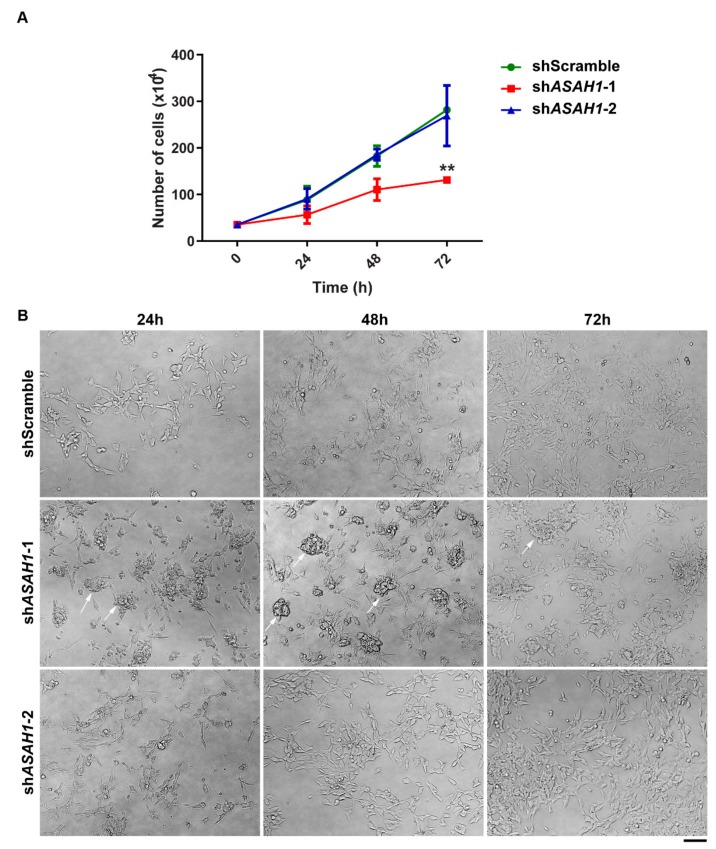
sh*ASAH1*-1 SH-SY5Y cell line displays lower proliferation rate and morphological alterations. (**A**) Growth curves of three different SH-SY5Y cell lines, shScramble (green line), *shASAH1*-*1* (red line) and *shASAH1*-*2* (blue line), were constructed by scoring trypan blue-negative cells at 0, 24, 48, and 72 h after cell seeding. Data are expressed as the mean ± SD of two independent experiments. ** *p* < 0.005 compared to shScramble cells (two-way ANOVA analysis) (**B**) Representative pictures of cell growth and morphology using phase contrast microscopy. The scale bar represents 40 μm for all panels.

Observation of differential silencing efficiencies prompted us to analyze both sh*ASAH1*-1 and sh*ASAH1*-2 cells for changes in in vitro cell proliferation and morphology compared to shScramble cells, to allow an inference on the dose effect of AC. To this end, proliferation curves were generated (Figure 2A) and cell images were obtained (Figure 2B) over time (24 h, 48 h, and 72 h) after seeding identical cell numbers for all three cell lines. The proliferation rate of sh*ASAH1*-1 cells was considerably reduced compared to shScramble cells, with significant reduction by 50% at 72 h (Figure 2A, red line), whereas proliferation for sh*ASAH1*-2 cells (blue line) was comparable to that for shScramble cells (green line). Moreover, sh*ASAH1*-1 cells showed alterations in cell morphology; cells were more spherical and formed foci (Figure 2B, arrows) compared to shScramble cells, which showed an elongated shape with short neurite outgrowths (Figure 2B). The greater knockdown efficiency and the clear biological impact of sh*ASAH1*-1 on SH-SY5Y cells prompted us to exclude sh*ASAH1*-2 cells and focus on the stable sh*ASAH1*-1 cell line, in the following designated as *ASAH1*^KD^, for further investigation.

### 2.2. AC Reduction Induces Cell Cycle Arrest at G1/S Phase and Apoptosis

**Figure 3:**
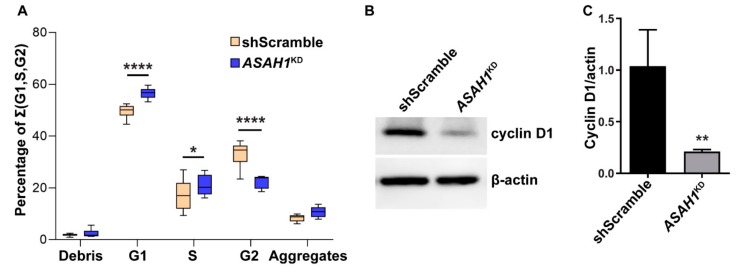
Acid ceramidase (AC) depletion induces G1/S cell cycle arrest of *ASAH1*^KD^ cells. (**A**) Cell cycle progression was assessed using propidium iodide and flow cytometry analysis. The graph gives percentages of G1, S, and G2 as a total of 100% with relative percentages of debris and aggregates, in *ASAH1*^KD^ and shScramble cells. * *p* = 0.02, **** *p* < 0.0001 compared to shScramble (*n* = 6, two-way ANOVA of percentages). (**B**) Representative Western blot results showing decreased expression levels of cyclin D1 in *ASAH1*^KD^ cells. β-actin was used as loading control. (**C**) Graph of densitometric measurements of cyclin D1 expression normalized with β-actin internal control, as illustrated in panel B. Data are expressed as mean ± SD (*n* = 3, ** *p* < 0.006, Student *t*-test).

We then performed flow cytometry and Western blot analyses in *ASAH1*^KD^ cells in order to dissect the observed effect of AC depletion on cell physiology. Flow cytometry was used to investigate changes in the distribution of the cell cycle phases (Figure 3A) and showed that only 22.5% of *ASAH1*^KD^ cells entered the S phase and 56.6% were retained in G1, compared to shScramble cells, of which 33.1% and 49.6% were in S and G1 phase, respectively. The results were substantiated by Western blot analysis using cyclin D1 as a marker of cell cycle progression at the G1/S-phase transition, which was detected significantly reduced by 80% in *ASAH1*^KD^ cells compared to shScramble cells (Figure 3B,C). Combination of flow cytometry and immunoblot data indicate that AC depletion stalls cell cycle progression and thus cell proliferation by interference with transition from G1 to S phase.

**Figure 4:**
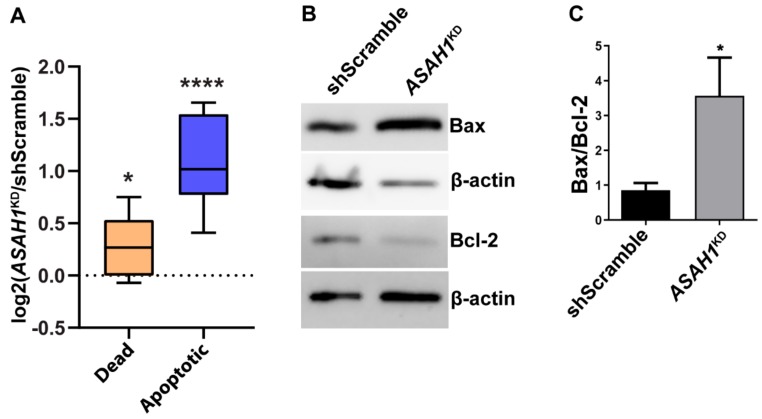
AC depletion induces apoptosis in *ASAH1*^KD^ cells. (**A**) Box-and-whisker diagram of individual log2-transformed percentage ratios of *ASAH1*^KD^ cells to shScramble for cell death and apoptosis, respectively. Data points above 0 indicate a percentage increase for *ASAH1*^KD^ cells. **** *p* = 0.002 for apoptosis and * *p* = 0.0264 for cell death (*n* = 9, one-sample t-test with multiple testing correction). (**B**) Representative Western blot results showing an increase of pro-apoptotic marker Bax and a decrease of anti-apoptotic marker Bcl-2 in *ASAH1*^KD^ cells. β-actin was used as loading control. (**C**) Ratio of densitometric measurements of Bax/Bcl-2 expression normalized with β-actin as internal control, as illustrated in panel B. Data are expressed as mean ± SD (*n* = 3, *p = 0.02, Student *t*-test).

To investigate whether AC depletion in *ASAH1*^KD^ cells also induced apoptosis, we performed flow cytometry for cell viability staining. *ASAH1*^KD^ cells had 2.1-fold increased apoptosis and significantly elevated cell death compared to shScramble cells (Figure 4A). Elevated apoptosis was also confirmed by Western blot analysis for the pro-apoptotic marker Bax and the anti-apoptotic marker Bcl-2 (Figure 4B). Bax levels were increased and Bcl-2 levels decreased in *ASAH1*^KD^ cells, (Figure 4B), resulting in a significantly, 3.5-fold increased Bax/Bcl-2 ratio compared to shScramble cells (Figure 4C) and confirming induction of apoptosis by AC depletion.

Combined, the above flow cytometry and immunoblot results for both, cell cycle and apoptotic markers, indicate that the observed reduction in *ASAH1*^KD^ proliferation is attributable to both cell cycle arrest and elevated apoptosis.

### 2.3. AC Depletion Leads to a Diffuse Distribution Pattern of Lysosomes

**Figure 5:**
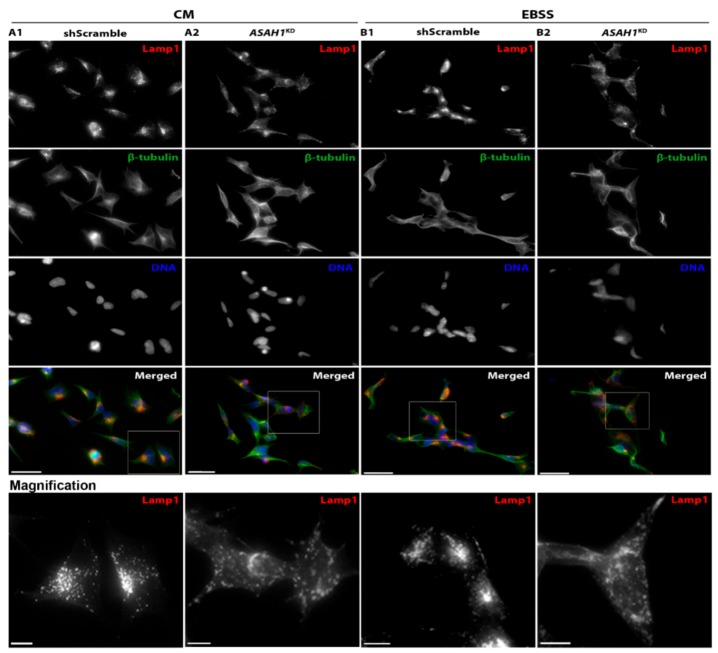
AC depletion alters the distribution of lysosomes. Immunofluorescence microscopy images of (**A1**) shScramble cells show the normal distribution of lysosomes and (**A2**) *ASAH1*^KD^ cells show a more diffuse pattern of lysosomes, under normal conditions (control medium, CM). (**B1**) shScramble and (**B2**) *ASAH1*^KD^ cells under starvation conditions (EBSS medium, for 2 h) show differential lysosome distribution, with juxtanuclear location and continued diffuse distribution, respectively. Scale bars in merged images represent 40 μm. For higher magnification images the scale bars represent 20 μm. Lysosomes were stained with LAMP1 antibody (red in merged images), cytoskeleton with β-tubulin (green in merged images) and the nuclei with Hoechst dye (blue in merged images).

To investigate the effect of AC depletion on distribution of lysosomes, we labelled the lysosomes with LAMP1 marker and the cytoskeleton with β-tubulin marker. In shScramble cells, lysosomes were distributed in the cytoplasm with mostly perinuclear detection (Figure 5A1), whereas in *ASAH1*^KD^, lysosomes were more diffuse throughout the cytoplasm and neurite outgrowths (Figure 5A2). Upon starvation, lysosomes were concentrated in the juxtanuclear region in the shScramble cells (Figure 5B1), consistent with previous studies [20], whereas lysosomes in *ASAH1*^KD^ cells continued to remain disperse (Figure 5B2). AC depletion thus abolished the ability of LAMP1-positive lysosomes to redistribute to the juxtanuclear region after starvation.

**Figure 6:**
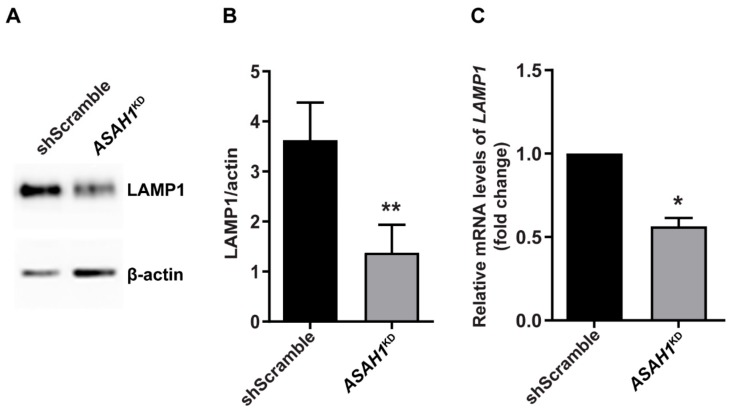
Decrease of protein and mRNA levels of LAMP1 in *ASAH1*^KD^ cells. (**A**) Representative immunoblot using antibodies against LAMP1 and β-actin. (**B**) Levels of LAMP1 normalized for β-actin expression. Data are expressed as mean ± SD (*n* = 4, ** *p* < 0.003, Student *t*-test). (**C**) Relative mRNA expression levels of LAMP1 in *ASAH1*^KD^ cells to shScramble cells after normalization with GAPDH. Data are represented as the mean ± SEM of two independent triplicate experiments (* *p* = 0.02, Student *t*-test).

To evaluate any potential change in LAMP1 levels, we performed Western blot analysis. As shown in Figure 6A,B, a 62% decrease in LAMP1 protein levels was observed in *ASAH1*^KD^ cells compared to shScramble cells. This decrease of LAMP1 was also reflected at the transcript level for *LAMP1*, for which RT-qPCR was decreased by 1.8-fold in *ASAH1*^KD^ cells compared to shScramble cells (Figure 6C).

### 2.4. ASAH1^KD^ Cells Display Shorter Neurites and Fewer Branches Per Neurite

**Figure 7:**
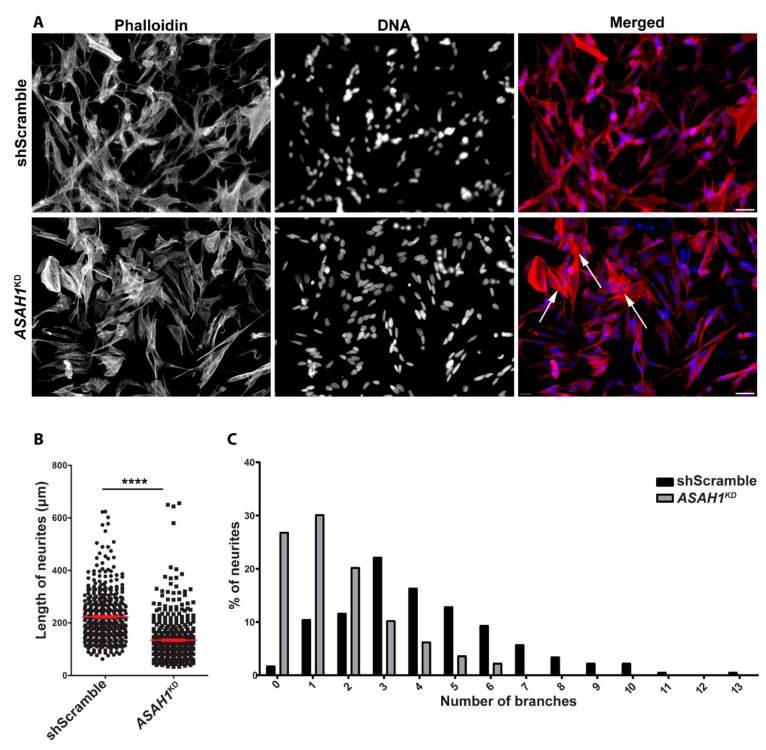
*ASAH1*^KD^ cells display short neurites and fewer branches per neurite. (**A**) Immunofluorescence microscopy images of differentiated shScramble and *ASAH1*^KD^ cells stained with the phalloidin (Alexa Fluor^®^ 568 dye, red). Nuclei were stained with Hoechst dye (blue). The scale bars correspond to 40 μm. White arrows show cells with stress fibers. (**B**) Length of neurites (μm) per cell. The mean neurite length was significantly lower in *ASAH1*^KD^ cells compared to shScramble cells (**** *p* < 0.0001, Student *t*-test). (**C**) Distribution of the number of branches per neurite (*p* < 0.0001, Chi-square test).

To investigate the effect of AC depletion on neuronal differentiation and neurite outgrowth, shScramble and *ASAH1*^KD^ cells were differentiated with retinoic acid, and the neuronal plasticity marker GAP-43 was used to evaluate the differentiation (Figure S1). To evaluate the actin cytoskeleton organization and neurite outgrowths, phalloidin staining was used. *ASAH1*^KD^ cells showed alterations in cellular morphology, being more rounded than shScramble cells, forming foci, displaying a limited number of neurites, and losing their ability to display intercellular connections and neuronal networks (Figure 7A). In *ASAH1*^KD^ cells, there were more cells with stress fibers (Figure 7, white arrows).

Quantification of neurite length showed that AC-depleted cells had shorter neurites compared to shScramble cells (Figure 7B). The mean length of neurites of AC-depleted cells (*n* = 450) was 134.5 μm compared to 223 μm of shScramble cells (*n* = 476), a 40% decrease. Moreover, quantification of the branches per neurite showed that the frequency distribution of branches in AC-depleted cells was significantly different from that of shScramble cells (Figure 7C), with a shift towards lower branch numbers. In AC-depleted cells, 26.9% of neurites had no branch, 30.2% had only one branch, 20.3% and 10.3% had two or three branches, respectively, and only 12.3% had ≥ 4 branches, with a maximum of six branches per neurite. In contrast, in shScramble cells, only 1.8% of neurites had no branch, 10.5% had only one branch and the majority of the shScramble cells had three (22.2%), four (16.4%), and five (12.9%) branches. Of note, 15.1% of shScramble cells displayed neurites with 7 to 13 branches, which were altogether absent from *ASAH1*^KD^ cells (Figure 7C).

The results indicate that AC-depleted cells have a limited ability to generate and extend neurites and that AC is essential for normal neurite development.

### 2.5. AC Depletion Causes Significant Up-Regulation of the RHOA Transcript

**Figure 8:**
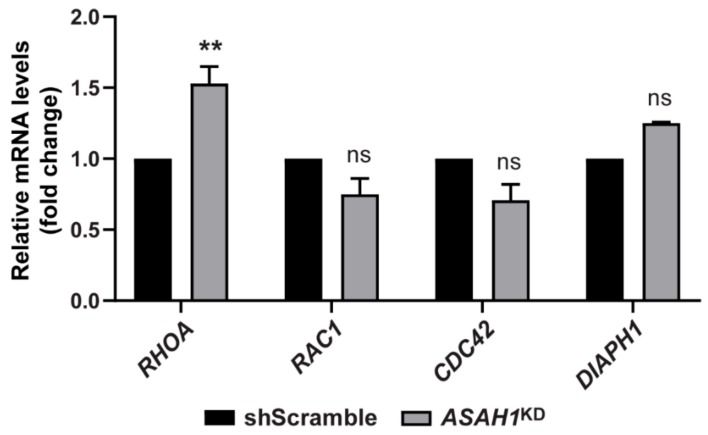
mRNA expression of the Rho GTPase family members. The graph represents the mRNA levels of *RHOA*, *RAC1*, *CDC42*, and *DIAPH1* genes in *ASAH1*^KD^ cells relative to shScramble cells after normalization with the endogenous *GAPDH* gene. Values represent the mean ± SEM of two independent triplicate experiments. An ** indicates significance (*p* = 0.0026, two-way ANOVA analysis).

To investigate if the different morphological profile of *ASAH1*^KD^ cells could be due to alterations in the expression of the Rho GTPase genes, we performed RT-qPCR, using specific primers against four members of this family; *RHOA*, *RAC1*, *CDC42*, and *DIAPH1*. These members of the Rho family of GTPases have been shown to regulate many aspects of cytoskeleton dynamics [21], cell cycle progression [22], neuronal morphology [23] and lysosome positioning [24,25]. AC depletion significantly increased the mRNA levels of *RHOA* by 1.53-fold (Figure 8) in ASAH1^KD^ cells, but other GTPases’ levels were not significantly changed (Figure 8).

### 2.6. AC Depletion Leads to Changes in Sphingolipid Content

The intracellular levels of various species of ceramide (Cer), dihydroceramide (dhCer), sphingomyelin (SM), hexosylceramide (HexCer), ceramide-1-phosphate (Cer-1P), and sphingosine (Sph), dihydrosphingosine (dhSph), sphingosine-1-phosphate (Sph-1P), and dihydrosphingosine-1-phosphate (dhSph-1P) in *ASAH1*^KD^ and shScramble stable cell lines were quantified using high-performance liquid chromatography mass spectrometry (HPLC-MS/MS).

**Figure 9:**
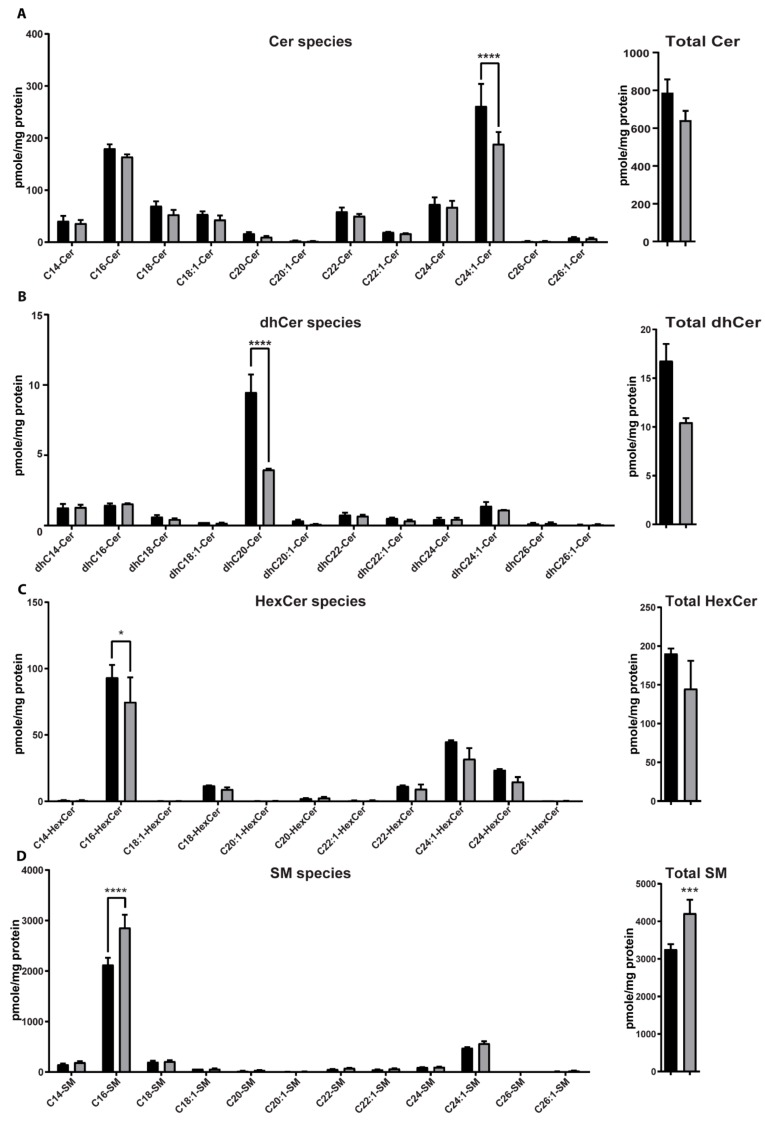

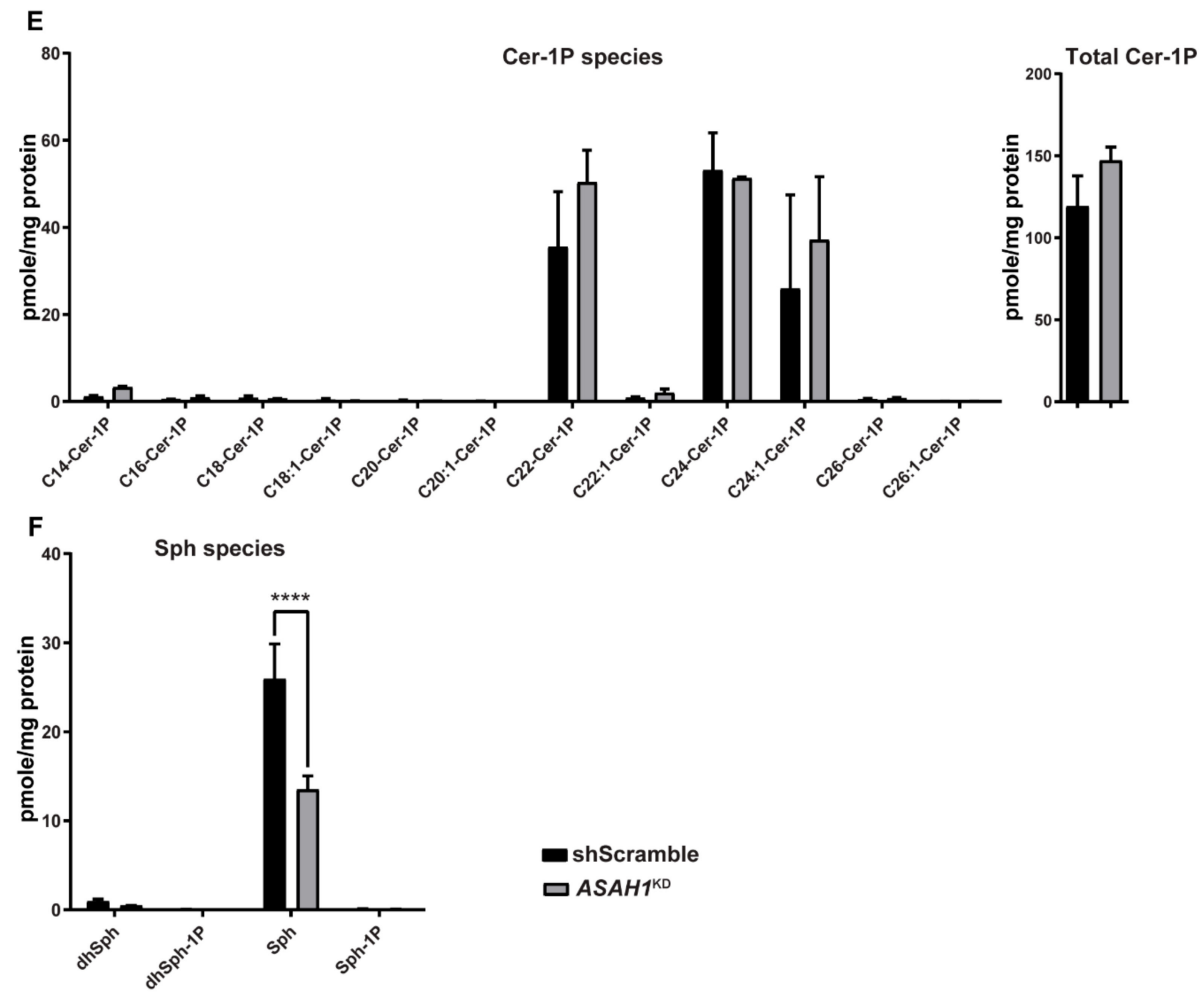
AC depletion causes changes in sphingolipid content. HPLC-MS/MS quantification analysis of the intracellular levels of different subspecies of medium- and longer-chain sphingolipids in shScramble (black color) and *ASAH1*^KD^ (grey color) cells: (**A**) ceramide (Cer), (**B**) dihydroceramide (dhCer), (**C**) hexosylceramide (HexCer), (**D**) sphingomyelin (SM), (**E**) ceramide-1-phosphate (Cer-1P) and (**F**) dihydrosphingosine (dhSph), dihydrosphingosine-1-phospate (dhSph-1P), sphingosine (Sph) and sphingosine-1-phospate (Sph-1P). The different subspecies are represented with the chain length and number of double bonds. To the right of panel A through E, the total amount of the particular sphingolipid is presented. Results are expressed as mean ± SD in pmoles/mg protein (*n* = 2, * *p* = 0.01, *** *p* < 0.001 and **** *p* < 0.0001, two-way ANOVA).

Total Cer levels were slightly decreased and no significant differences were observed among the different subspecies of Cer, except for a significant 27.8% decrease in the long-chain unsaturated C24:1 Cer in *ASAH1^KD^* compared to shScramble cells (Figure 9A). The total lipid levels of dhCer (Figure 9B) and HexCer (Figure 9C) were also slightly decreased in *ASAH1*^KD^ cells compared to shScramble cells. Regarding the different subspecies of dhCer, the dhC20-Cer was found to be the most abundant dhCer in both, *ASAH1*^KD^ and shScramble cells, with a significant 58.8% decrease in *ASAH1*^KD^ cells (Figure 9B). Additionally, C16, C24:1 and C24 species of HexCer were decreased in *ASAH1*^KD^ cells compared to shScramble cells, but only C16-HexCer specie displayed a significant decrease by 19.9% (Figure 9C). In contrast, total SM levels were significantly increased by 29.5% in *ASAH1*^KD^ cells, with a significant 34.5% increase of sphingomyelin C16:0 among SM subspecies (Figure 9D). Total levels of Cer-1P were slightly increased in *ASHA1*^KD^ compared to shScramble cells with C22 and C24:1 species as key contributors to this elevation (Figure 9E). Regarding Sph molecular species, Sph was significantly decreased by 47.9% in *ASAH1*^KD^ cells compared to shScramle cells, whereas dhSph, dhSph-1P, and Sph-1P were almost undetectable in both cell lines (Figure 9F).

### 2.7. AC Depletion Causes Alteration in Genes that Control Cer Homeostasis

**Figure 10:**
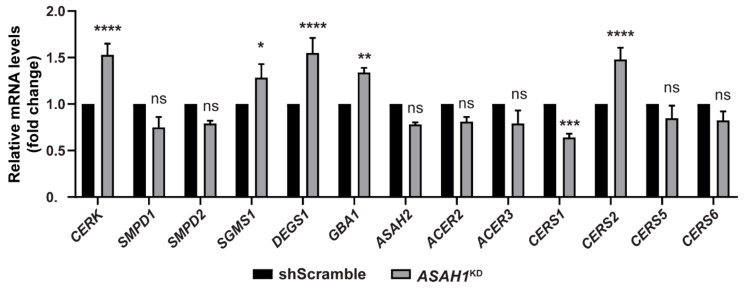
Altered mRNA levels of genes expressing enzymes of ceramide metabolism in *ASAH1*^KD^ cells. The graph represents the mRNA levels of *CERK* (Cer kinase), *SMPD1* and *SMPD2* (acid and neutral sphingomyelinase), *SGMS1* (SM synthase 1), *DEGS1* (delta 4-desaturase, sphingolipid 1), *GBA* (glucosylceramidase beta), *ASAH2* (neutral ceramidase), *ACER2* and *ACER3* (alkaline ceramidases 2 and 3) and ceramide synthases (*CERS1*, *CERS2*, *CERS5*, and *CERS6*) sphingolipid genes in *ASAH1*^KD^ cells relative to shScramble cells after normalization with the endogenous *GAPDH* gene. Values represent the mean ± SEM of three independent triplicate experiments. Asterisks indicate statistically significant (* *p* < 0.05, ** *p* < 0.01, *** *p* < 0.001, and **** *p* < 0.0001 significant, two-way ANOVA analysis).

**Figure 11:**
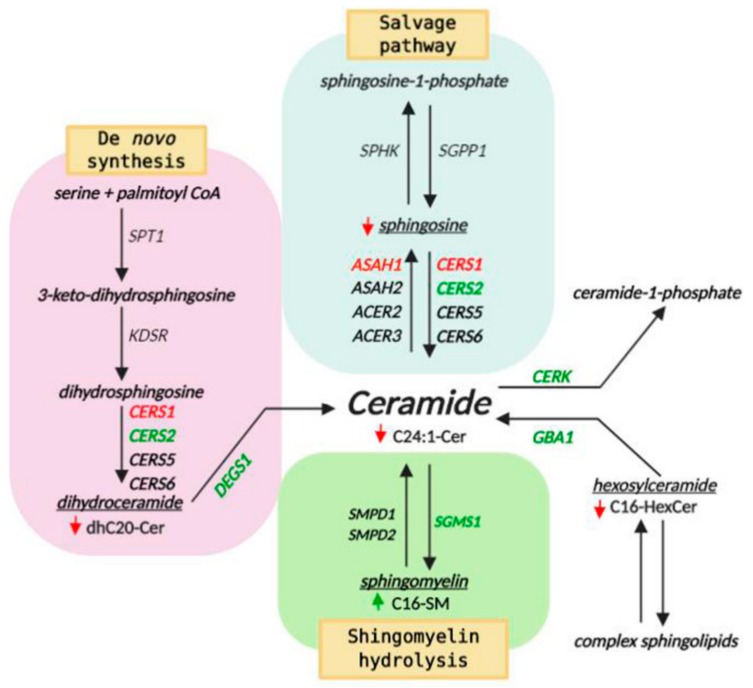
Altered sphingolipid and gene transcript levels in *ASAH1*^KD^ SH-SY5Y cells. Illustration of the three major metabolic pathways of ceramide biosynthesis: the de novo pathway (pink background), the salvage pathway (light blue background) and the sphingomyelin hydrolysis pathway (green background). Green arrows indicate an increase and red arrows indicate a decrease in the intracellular sphingolipid levels. Genes encoding enzymes involved in these pathways are shown in *italics*, with colors indicating significant up-regulation (green) and down-regulation (red) of corresponding mRNA. *ASAH1*, lysosomal ceramidase; *ASAH2*, plasma membrane ceramidase; *ACER2*, golgi ceramidase; *ACER3*, endoplasmic reticulum and golgi ceramidase; *CERS1, 2, 5, 6*, ceramide synthases; *DEGS1*, dihydroceramide desaturase; *CERK*, ceramide kinase; *SGMS1*, sphingomyelin synthase 1; *SMPD1*, lysosomal sphingomyelin phosphodiesterase 1; *SMPD2*, neutral sphingomyelin phosphodiesterase 2; *GBA1*, glucosylceramidase beta 1; *SPT1*, serine palmitoyltransferase; *KDSR*, 3-ketodihydrosphingosine reductase; *SPHK*, sphingosine kinase; *SGPP1*, sphingosine-1-phosphate phosphatase 1.

Taking in consideration the alteration of sphingolipid content in the *ASAH1*^KD^ stable cell line, we next examined the transcription of genes involved in Cer metabolism, in order to evaluate the effect of AC depletion on the transcript levels of these genes. Reduced AC expression affects the transcript levels of several of Cer-related genes (Figure 10), including significant mRNA upregulation for ceramide kinase (*CERK*), delta 4-desaturase, sphingolipid 1 (*DEGS1*) and ceramide synthase 2 (*CERS2*), by 1.5-fold each. Sphingomyelin synthase 1 (*SGMS1*) and glucosylceramidase beta (*GBA*) were also significantly upregulated, by 1.3-fold each. In contrast, the expression levels of ceramide synthase 1 (*CERS1*) were 1.6-fold decreased compared to shScramble levels. The mRNA levels for two sphingomyelinases, acid (*SMPD1*) and neutral (*SMPD2*), as well as ceramide synthases (*CERS5* and *CERS6*) and neutral (*ASAH2*) and alkaline (*ACER2* and *ACER3*) ceramidases were not significantly changed. The results of Section 2.6 and Section 2.7 are summarized in Figure 11.

## 3. Discussion

In the current study, we established stable knockdown SH-SY5Y cell lines expressing short hairpin RNAs (shRNA) against *ASAH1* after lentiviral transduction and used them to determine the functional significance of AC in human neuronal cells. Moderate reduction of *ASAH1* transcript, protein and activity levels in the sh*ASAH1*-2 cell line had no apparent effect on cell morphology and proliferation, whereas efficient reduction in a second cell line, sh*ASAH1*-1 (*ASAH1*^KD^), significantly impaired both parameters. *ASAH1*^KD^ cells had a limited number of neurites and displayed fewer intercellular connections than the shScramble after differentiation. Additionally, *ASAH1*^KD^ cells had significantly shorter neurites and markedly fewer branches per neurite, compared to shScramble cells. These results are in agreement with those of Zhou et al., who showed that knockdown of the *ASAH1* ortholog in zebrafish, using a morpholino antisense oligonucleotide, leads to increased apoptosis in the spinal cord, and to a significant loss of axonal branching in motor neurons [3]. Shorter axons of motor neurons but with a higher number of terminal branches have also been observed in zebrafish after knockdown of the *GBA2* orthologous gene, which encodes the non-lysosomal glucosylceramidase, an upstream enzyme in the same sphingolipid pathway [26].

In neurons, the Rho GTPase family members are important regulators of neuronal morphology and polarity, playing a critical role in axonal growth, cone dynamics and neurite branching, by controlling F-actin and microtubule cytoskeleton dynamics [23]. The best-known members of this family are the RhoA, Rac1 and Cdc42 proteins, which demonstrate opposing functions. We found that knockdown of AC caused significant upregulation of RhoA mRNA and downregulation of Rac1 and Cdc42 mRNAs. RhoA regulates the formation of actin stress fibers and focal adhesions [21], which might explain the elevated number of *ASAH1*^KD^ cells containing stress fibers. The other two members of the Rho GTPase family, Rac1, and Cdc42, are involved in the formation of lamellipodia and filopodia, respectively [21]. The downregulation of these corresponding transcripts might explain the rounded phenotype and the foci formation in *ASAH1*^KD^ cells. This phenotype was also observed in cells silenced for PHF8, a histone demethylase associated with X-linked mental retardation [27]. In PHF8 knockdown cells, these Rho GTPase members were also downregulated, and cells had a limited ability to generate neurites [27]. Moreover, expression of a dominant-negative mutant of Rac1 in primary rat spinal motor neurons caused apoptosis of motor neurons and reduction of axon outgrowth [28]. Importantly, an upregulation of RhoA and downregulation of Cdc42 have been reported in SMN knockdown PC12 cells [29] and an enhanced activation of RhoA/ROCK pathway in the spinal cord of a SMA mouse model [30], contributing in the development of defects in neuritogenesis.

Rho GTPases are also involved in gene transcription [31], cell cycle progression [22] and neuronal survival [32,33]. RhoA activation and its downstream effectors promote neuronal death (activation of pro-apoptotic proteins), whereas Rac1 and its downstream effectors promote neuronal survival (activation of pro-survival and inhibition of pro-apoptotic proteins) [34,35]. The balance between these two distinct signal pathways is essential to the survival of neurons [23]. We have shown that *ASAH1*^KD^ cells have elevated levels of the pro-apoptotic Bax protein and lower levels of the pro-survival Bcl-2 protein compared to shScramble cells. The induction of apoptosis was also confirmed by flow cytometry. Elevated apoptosis in *ASAH1*^KD^ cells can be explained by the combination of upregulation of RhoA and downregulation of Rac1, as observed in *ASAH1*^KD^ cells.

Additionally, we demonstrated that *ASAH1*^KD^ cells had a reduced proliferation rate compared to shScramble cells, expressed lower levels of the cyclin D1 protein and were arrested at the G1/S phase of the cell cycle. It is well known that Rho GTPases also act to regulate transcription and translation of cyclin D1 and therefore contribute to G1 progression [36,37].

AC is considered a lysosomal enzyme involved in the degradation of Cer at acidic pH [38]. Lysosomes are highly dynamic structures, moving bi-directionally in a “stop-and-go” manner controlled by microtubule-based motor proteins, kinesins (anterograde movement) and dynein (retrograde movement), and the GTPase effector pairs [39,40,41]. Lysosomes are scattered throughout the cytoplasm, but are mostly concentrated in the perinuclear region surrounding the microtubule-organizing center [42]. In this study, we demonstrated that the positioning of LAMP1-positive lysosomes changed after knockdown of AC. LAMP1-positive lysosomes were distributed throughout the cytoplasm and neurite outgrowths in undifferentiated *ASAH1*^KD^ cells, whereas in undifferentiated shScramble cells, most were concentrated in the perinuclear region and few scattered throughout the cytoplasm. The perinuclear lysosomes are relatively immobile, highly acidic (pH 4.5–5.5) and have high degradative activities [42,43]. In contrast, peripheral lysosomes are more mobile, less acidic (pH > 5.5) and with lower degradative activities [43]. We speculate that the lysosome distribution towards the cell periphery in *ASAH1*^KD^ cells is due to upregulation of RhoA. In line with this hypothesis, transfection of the dominant active RhoA into rat hepatoma cells was shown to induce the peripheral distribution of cathepsin D and of LIMPI-positive lysosomes, and the formation of stress fibers [24,25].

Movement of lysosomes towards the cell periphery is also observed in cancer cell lines and is required for cancer invasion, migration, and metastasis [24,44]. In this case, lysosomal exocytosis occurs, leading to the secretion of acidic hydrolases and metalloproteinases that are required for degradation of the extracellular matrix to promote metastasis of cancer cells [45,46]. Recently, inhibition of AC was found to cause suppression of the interaction between lysosomes and the multivesicular body, and hence the increase of exosome release from podocytes, highlighting the role of AC in TRPML1 channel-mediated Ca^2+^ release [47]. Moreover, Li et al. demonstrated that different sphingolipids had differential effects on the TRPML1 channel, specifically that Cer had no effect, whereas Sph was found to enhance and SM to inhibit TRPML1 channel activity [47].

Changes in lysosome distribution have also been observed in some other lysosomal storage disorders (LSDs) that affect the central nervous system, but, interestingly, lysosomes in those LSDs are clustered in the juxtanuclear region. Mislocalization of the lysosomes is observed at an early stage in mouse models of Gaucher disease (GD) displaying neurological pathology [48]. A generation of several large vesicles and linear structures in the perinuclear area was observed in these mouse models [48]. Additionally, clustering of abnormal lysosomes in the perinuclear area was observed in ceroid lipofuscinosis type 3 (CLN3) [49] and mucolipidosis type IV (ML-IV) [50]. Precisely how defects in acidic hydrolases cause alterations in lysosome positioning is still unclear.

We have also shown that expression of mRNA and protein levels of LAMP1 were significantly decreased in *ASAH1*^KD^ cells, down to half of that found in shScramble cells. Low LAMP1 protein levels may reflect changes in the number or size of lysosomes. Similar results were found in mouse models of GD, where the number of LAMP1-positive vesicles was significantly decreased [48].

Lysosomal dysfunction and impairment of endolysosomal trafficking has also been reported to contribute to the pathology of several neurodegenerative diseases including Parkinson’s disease, Alzheimer’s disease and amyotrophic lateral sclerosis [51,52,53,54,55]. For example, the expression of mutant forms of leucine-rich repeat kinase 2 (LRRK2) [56] or huntingtin [57], causes perinuclear clustering of lysosomes.

The distribution of lysosomes inside the cytoplasm is regulated by a variety of stimuli. Acidification of the cytoplasm can cause dispersal of the lysosomes from the perinuclear region to the periphery of the cell [58]. There is also the opposite effect, the perinuclear clustering of the lysosomes under specific conditions such as starvation [20], drug-induced apoptosis [59], and oxidative stress [60]. In this study, we induced starvation using EBSS media, in order to investigate related lysosomal distribution by immunofluorescence. As expected, in shScramble cells, lysosomes were clustered at the juxtanuclear area, as nutrient starvation inhibits mTORC1 and induces movement of the autophagosomes and lysosomes toward the juxtanuclear region, thereby facilitating organelle fusions [20]. Importantly, in *ASAH1*^KD^ cells, LAMP1-positive lysosomes continued to distribute throughout the cytoplasm, indicating impairment of lysosomal translocation and re-organization.

AC catalyzes the hydrolysis of Cer to Sph and free fatty acids at acidic pH, but also synthesizes Cer at neutral pH [24], so that it is considered a “rheostat” for maintaining the balance between the levels of Cer and Sph, two important bioactive sphingolipids emerging as key regulators of diverse cellular processes, including growth, differentiation, apoptosis and autophagy [61]. In this study, we have shown that AC depletion had various effects on sphingolipid molecules levels and on the transcription of genes that are involved in Cer metabolism.

Sph levels were significantly decreased, as was expected, since Cer degradation by ceramidases is the only source of Sph inside the cells [62]. Contrary to expectations, total Cer levels were slightly decreased instead of increased in *ASAH1*^KD^ cells compared to shScramble cells. This finding is consistent with the results of Zhou et al., who showed that 75% reduction of AC activity in morphants compared to control zebrafish did not affect Cer content [3]. It has also been reported that mildly affected FD patients do not show accumulation of Cer in liver, lung or brain [63]. Lucki et al. also showed that the total Cer levels were unchanged in *ASAH1*^KD^ H295R human adrenocortical cells [64].

In contrast, Cer accumulation was documented in cell models, in which AC was knockdown or inhibited by specific inhibitors [65,66,67] or in mouse models [7,68,69,70]. Particularly, different Cer subspecies were elevated or decreased depending on the cell line or tissue studied. More recently, Yu et al. using the *Asah1*^P361R/P361R^ knock-in mouse model showed alterations to various sphingolipids and altered gene transcription within the sphingolipid pathway in hepatocytes [69]. The observed discrepancy in the sphingolipid content between different experimental works may reflect cell-specific enzymatic and sphingolipid profiles. Therefore, the role of Cer accumulation in disease pathogenesis is still unclear. Cells might employ alternative mechanisms to maintain total Cer at a constant level despite depletion of AC. Cer is also degraded by other ceramidases that have different localization within the cells and different substrate specificity [71]. However, we have shown that the mRNA levels of the neutral ceramidase (*ASAH2*) and the two alkaline ceramidases (*ACER2* and *ACER3*) were unchanged in *ASAH1*^KD^ cells compared to shScramble cells, indicating that other ceramidases are not upregulated to maintain Cer catabolism.

Moreover, we found that the mRNA levels of the CerS5 and CerS6 enzyme isoforms were unchanged, whereas the CerS1 and CerS2 transcripts were significantly downregulated and upregulated, respectively, in *ASAH1*^KD^ cells. CerS1 is mainly expressed in brain neurons and spinal cord and metabolizes long-chain C18-Cer [72]. CerS2 is ubiquitously expressed, but more in myelinating oligodendrocytes, and is responsible for the production of very long-chain (C22–C26) Cer [73]. Mutations in both enzymes lead to progressive myoclonic epilepsy [74,75], whereas specific mutations in the *ASAH1* gene were found to cause spinal muscular atrophy with progressive myoclonic epilepsy [3,76,77,78,79], suggesting a link between dysregulation of sphingolipid metabolism and epilepsy.

The levels of unsaturated C24:1-Cer subspecies were significantly decreased in *ASAH1*^KD^ cells, although the mRNA levels of CerS2 were significantly upregulated. CerS2 is responsible for the synthesis of unsaturated very long-chain C24:1-Cer, and its mRNA levels were inversely correlated to the levels of this lipid. The reason for this discrepancy is not clear, and the specific role of AC in the regulation of sphingolipid acyl-chain composition still remains to be explored.

Additionally, we have shown that the dhC20-Cer and C16-HexCer subspecies were significantly decreased. Based on our results, this reduction may be partially due to upregulation of *DEGS1*, which encodes an enzyme that converts dhCer to Cer, and lysosomal GBA, which degrades GluCer to Cer. The transcripts of *CERK*, which encodes an enzyme responsible for conversion of Cer to Cer-1P, were also found to be upregulated in *ASAH1*^KD^ cells, and this may explain the fact that total Cer-1P levels, and particularly the C22 and C24:1 subspecies, were slightly increased. Importantly, total SM levels were found significantly increased, with C16-SM being significantly elevated. The transcripts of *SGMS1*, the protein product of which synthesizes SM from Cer, were found significantly increased, and the transcripts of *SMPD1* and *SMPD2*, the products of which degrade SM to Cer at acid and neutral pH, respectively, were both found slightly downregulated in *ASAH1*^KD^ cells. The combination of the action of these three enzymes can explain the increase of C16-SM levels in *ASAH1*^KD^ cells. Additionally, it was previously found that AC interacts with acid sphingomyelinase and that its upregulation causes upregulation of acid sphingomyelinase [80], an observation tying in with increased SM levels upon AC depletion, as observed in our study.

Plasma membranes, especially of neurons, contain specific microdomains, the so-called lipid rafts. In neurons, lipid rafts are present in the axonal plasma membrane and are important for neuronal function [81]. These domains are rich in sphingolipids, especially SM and cholesterol [82], and act as a platform for glycosylphosphatidylinositol (GPI)-anchored proteins and signal transduction molecules [83]. Many of these molecules, such as Src-family kinases and Rho GTPases, as well as the phosphoinositide PtdIns(4,5)P2 and PtdIns(3,4,5)P3 lipids, are involved in regulation of the actin cytoskeleton and adhesion [84]. Lipid rafts are important for growth factor signal transduction, vesicular trafficking, synaptic transmission and axon guidance [85]. Ceramides, composed mostly of long acyl chains, are present in ordered domains with SM and cholesterol [86] and can induce membrane fusion and vesicular transport [87]. Specifically, C24:1-Cer was shown to form with mono-unsaturated phosphatidylcholine gel-phase domains [88] and exhibits a unique thermotropic behaviour in the C16-SM domains that may affect their biological function [89]. We speculate that the change of sphingolipid composition that we observed in *ASAH1*^KD^ cells with increased C16-SM and decreased C24:1-Cer levels, induces a change in lipid packaging of rafts that influences the function of proteins like RhoA, thereby regulating cytoskeletal dynamics and signal transduction pathways.

## 4. Materials and Methods

See Supplementary Table S1 for sequences of oligonucleotide primers, Supplementary Table S2 for all antibodies used and Supplementary Table S3 for details regarding two-way ANOVA analysis.

### 4.1. Cell Culture and Differentiation

Undifferentiated human neuroblastoma SH-SY5Y cells (ECACC Sigma Aldrich, St. Louis, Mo, USA) were cultured in DMEM supplemented with 10% FBS, 1% penicillin/streptomycin and 1% Glutamax at 37 °C, 5% CO_2_. For differentiation, cells were grown for 4 days on poly-*D*-Lysine-coated coverslips (0.05 mg/mL) in DMEM medium supplemented with 10 μM all-trans-retinoic acid (Sigma Aldrich, St. Louis, MO, USA). Differences in morphology were evaluated on an IX73 inverted microscope (Olympus, Tokyo, Japan) by phase contrast light microscopy. For starvation, cells were incubated for 2 h with Earle’s Balanced Salt Solution (EBSS).

### 4.2. Generation of Stable ASAH1 Knockdown SH-SY5Y Cell Lines

For the establishment of stable *ASAH1* knockdown SH-SY5Y cell lines, the following shRNA-encoding pairs of oligonucleotides were annealed and ligated into the pLKO.1-TRC vector (Addgene plasmid #10878, deposited by David Root) [90] as recommended. shRNA1—forward 5′-ccggctgTTATTGACAGCGATATAActcgagTTATATCGCTGTCAATAAcagtttttg-3′ and reverse 5′-aattcaaaaactgTTATTGACAGCGATATAActcgagTTATATCGCTGTCAATAAcag-3′, shRNA2 forward 5′-ccggctgGAAGGCTCTCTCTCTTTCctcgagGAAAGAGAGAGAGCCTTCcagtttttg-3′ and reverse 5′-aattcaaaaactgGAAGGCTCTCTCTCTTTCctcgagGAAAGAGAGAGAGCCTTCcag-3′; target-specific sequences complementary to 3′UTR and 5′UTR regions of *ASAH1* (NM_177924.5) respectively, are shown in capital letters. A pLKO.1-scrambled shRNA vector (Addgene plasmid #1864, deposited by David Sabatini) [91], was used as control for knockdown. The packaging plasmid psPAX2 and envelope protein plasmid pMD2.G (Addgene plasmids #12259 and #12260, respectively, deposited by Didier Trono) [92], were used for the production of lentiviral particles in HEK293T cells (Invitrogen, Carlsbad, USA). psPAX2 and pMD2.G along with either shRNA1 or shRNA2 or scramble shRNA were transfected into HEK293T cells using calcium phosphate co-precipitation. The cell culture supernatant containing lentiviral particles was collected and used to infect SH-SY5Y cells. Finally, cells stably expressing shRNA1 (sh*ASAH1*-1), shRNA2 (sh*ASAH1*-2), or scramble shRNA (shScramble) were selected with medium containing 2 μg/mL puromycin (InvivoGen, San Diego, CA, USA).

### 4.3. Cell Viability Calculation

To determine the rate of cell growth, equal volumes of cell suspension and 0.4% trypan blue (Sigma-Aldrich, St. Louis, MO, USA) were mixed, and the number of viable cells was determined using a hemocytometer (Hausser Scientific, PA, USA).

### 4.4. Neurite Outgrowth

To measure neurite outgrowth after differentiation, SH-SY5Y cells were seeded onto coverslips and then fixed with 4% paraformaldehyde (PFA) in PBS for 10 min at room temperature (RT). Images were acquired using an Olympus IX73 inverted microscope (Olympus Corporation, Tokyo, Japan) at a magnification of 20X. Neurite length was assessed using cellSens Dimension v.1.12 Software).

### 4.5. Reverse-Transcription Quantitative PCR (RT-qPCR)

Total RNA was isolated from stable knockdown SH-SY5Y cell lines using the RNeasy^®^ Midi kit (Qiagen, Hilden, Germany), and cDNA was generated from 1 μg of total RNA using the Protoscript^®^ M-MuLV II First Strand cDNA Synthesis Kit (New England Biolabs, Ipswich, MA, USA) according to the manufacturer’s instructions. Quantitative PCR was carried out using the ViiA7 Real-Time PCR system (Applied Biosystems, Foster City, CA, USA) and the amplifications were done using the KAPASYBR^®^ Fast qPCR Master mix according to the manufacturer’s instructions (Kapa Biosystems, Woburn, USA) and specific primers (Supplementary Table S1) for each gene (Eurofins Genomics, Ebersberg, Germany). All mRNA expression levels were normalized to glyceraldehyde 3-phosphate dehydrogenase (*GAPDH*) as housekeeping gene, and relative mRNA expression was calculated using the ΔΔCT method by REST-384, a Microsoft-Excel based software [93].

### 4.6. Enzyme Activity

Acid ceramidase activity was measured by fluorogenic assay according to Bedia et al. [94,95]. In brief, culture cells were collected by trypsinization, washed twice with PBS and re-suspended in 100 μL 0.2 M sucrose. After sonication and centrifugation, 25 μL of the resulting cell lysate (equivalent to 20 μg protein) or of 0.2 M sucrose (no-protein control sample) was added to make up 18.6 mM sodium acetate buffer (pH 4.5) and 20 μM Rbm-14-12 substrate in 100 μL total. After incubation at 37 °C for 3 h, the reaction was stopped by the addition of 50 μL of methanol and 100 μL of 2.5 mg/mL NaIO_4_ (in 100 mM glycine/NaOH buffer pH 10.6). After 2 h incubation in the dark, resulting fluorescence was quantified using a Synergy H1 microplate reader (λ_ex_ 360 nm, λ_em_ 446 nm) (BioTek, Winooski, Vermont, USA). To calculate the conversion of substrate by AC as nmol/hour/mg protein, a calibration curve with 4-methylumbelliferone standards was used. The activity of the enzyme in each sample was calculated according to the equation:

(1) $$Activity=\frac{\left(\frac{RFU\;value-B}{A}\right)*1000}{\left(volume\;of\;sample*sample\;concentration\right)}$$

where A and B were calculated from the standard curve, Y=A*X+B.

### 4.7. Flow Cytometry: Apoptosis and Cell Cycle Assays

Flow cytometry was performed using a CyFlow Cube 8 6-channel instrument (Partec/Sysmex). For the assessment of cell death and apoptosis, cells were stained with Yo-Pro-1 (Life Technologies, Thermo Fisher Scientific, Grand. Island, New York) and propidium iodide (Merck Millipore, Darmstadt, Germany) using an adaptation of published methods for viability staining [96]. Flow cytometry data for a minimum of 10^5^ gated events per sample (*n* = 9 per treatment, from three independent experiments of three samples per treatment each) were analysed in FCS Express 4.0 (De Novo Software). For cell-cycle analyses, cells were fixed with ethanol and stained with propidium iodide under treatment with RNase A (Qiagen) using an adaptation of published methods [97]. Curve fitting and quantification of cell distribution in G1, S, and G2 phases, including debris and aggregate distributions, was performed using ModFit LT V.5.0 (Verity Software House, Topsham, ME, USA), with an average reduced Chi square (RCS) value of 3.13 ± 0.82 across all samples (*n* = 6 per treatment).

### 4.8. Immunofluorescence Staining and Microscopy

Cells were fixed with 4% paraformaldehyde (PFA) in PBS for 10 min at RT and permeabilized with 0.1% Triton X-100 in PBS at RT or methanol for 10 min at −20 °C. Non-specific antibody binding sites were blocked with 3% bovine serum albumin in PBS containing 0.1% Tween™20 (PBST) for 1 h. Cells were then incubated for 1 h at RT with the appropriate primary antibodies diluted in 1% bovine serum albumin in PBST, were rinsed twice with PBST and incubated with the appropriate secondary fluorescence antibodies diluted in PBST for 1 h. Actin cytoskeleton and nuclei were stained with phalloidin (Alexa Fluor^®^ 568 dye, Invitrogen 1:200) for 30 min and with Bisbenzimidine Hoechst 33,342 (Sigma-Aldrich, St. Louis, MO, USA) for 1 min respectively. The coverslips were mounted with Dako fluorescence mounting media 53,023 (Agilent, Canada, USA) and analyzed with a Carl Zeiss Axiovert 200 M inverted fluorescence microscope.

### 4.9. Western Blot Analysis

Cells were harvested with trypsin, washed with PBS and lysed in lysis buffer (50 mM Tris pH 7.4, 150 mM NaCl, 1% Np-40, 0.5% Deoxycholate, 1X EDTA protease inhibitors). Following sonication, the protein concentrations were determined with the bicinchoninic acid (BCA) assay [98] using a standard curve of albumin, and the absorbance was measured at 562 nm by a Synergy H1 microplate reader (BioTek, Winooski, Vermont, USA). Proteins were resolved on a 12% SDS-PAGE gel and transferred onto a Porablot NCP nitrocellulose membrane (Macherey-Nagel, Duren, Germany). After blocking with 5% non-fat dried milk in PBST, the membrane was exposed to primary antibody diluted in 3% non-fat dried milk in PBST. After two washes in PBST, the membrane was incubated with HRP-conjugated secondary antibodies. Proteins were detected using the LumiSensor chemiluminescent HRP substrate (GenScript, Piscataway, USA) and images were obtained with a UVP BioSpectrum 810 imaging system (Fisher Scientific, Waltham, MA, USA). The digital images were quantified as optical density of band areas using *ImageJ* software and normalized to GAPDH or β-actin, which were used as the loading control for each lane, as indicated for each figure. GAPDH or β-actin was selected in each experiment depending on the molecular weight of the protein under investigation.

### 4.10. Quantification of Sphingolipids by HPLC/MS-MS

Quantitative analysis of intracellular sphingolipids; Cer, dhCer, HexCer, SM, Cer-1P, and sphingosine molecular species (dhSph, dhSph-1P, Sph, Sph-1P) was performed by Lipidomics Shared Resources—Analytical Unit (Medical University of South Carolina, Charleston, SC, USA) as previously described by Bielawski et al. (2009) [99], high-performance liquid chromatography mass spectrometry (HPLC-MS/MS) methodology. Briefly, 10^6^ cells of shScramble and *ASAH1*^KD^ cells were washed with PBS and collected by scraper. The protein concentration was determined and the lysates were lyophilized before lipid extraction and analysis. Results were expressed as pmoles of lipid/mg of total protein. Data are the mean of two independent experiments.

### 4.11. Statistical Analysis

GraphPad Prism 8 software (GraphPad Software, San Diego, CA, USA) was used for statistical analysis. Data were analyzed using the two-tailed Student’s *t*-test for pairwise comparison, one-way ANOVA for three independent groups comparisons, two-way ANOVA for multiple comparisons, the Chi Square test for distributions and the one-sample t-test with multiple testing correction for deviation of ratios from 1. An α = 0.05 was applied for statistical significance. For two-way ANOVA analysis the F and *p* values, the post-hoc test that was followed, as well as each *p* value for multiple comparisons for all experiments are provided in the Supplementary Table S3. Data were expressed as the mean ± SEM or the mean ± SD of at least two independent experiments as indicated in each figure legend.

## 5. Conclusions

In conclusion, our study has provided evidence that AC is an important regulator of neurogenesis, influencing survival, neurite length and the number of branches per neurite, as well as the positioning of LAMP1-positive lysosomes inside the cytoplasm. The defects observed were independent of Cer accumulation, since lipidomics analysis showed that the total levels of Cer subspecies were unchanged, if not decreased. While C24:1-Cer and C20-dhCer were decreased, C16-SM levels were significantly increased in *ASAH1*^KD^ cells, which combined with altered transcript levels for Rho GTPase family members and of enzymes involved in the metabolism of Cer, provide new pointers for therapy development of AC-related disorders. Of note, dysregulation of sphingolipid metabolism in *ASAH1*^KD^ cells might lead to changes in the composition of lipid rafts or even their abolishment, as a possible contributing factor, if not cause, for neuronal defects seen in AC-related neuropathology.

## Supplementary Materials

Supplementary materials can be found at https://www.mdpi.com/1422-0067/21/5/1607/s1.

## Abbreviations

AC	acid ceramidase
*ACER2*	plasma membrane ceramidase
*ACER3*	golgi ceramidase
*ASAH1*	lysosomal ceramidase
*ASAH2*	endoplasmic reticulum and Golgi ceramidase
Cer	ceramide
Cer-1P	ceramide-1-phosphate
CERK	ceramide kinase
CERS1,2,5,6	ceramide synthases
DEGS1	dihydroceramide desaturase
dhCer	dihydroceramide
dhSph	dihydrosphingosine
dhSph-1P	dihydrosphingosine-1-phospate
FD	Farber disease
GBA1	glucosylceramidase beta1
GluCer	glucosylceramide
HexCer	hexosylceramide
SGMS1	sphingomyelin synthase 1
SM	sphingomyelin
SMA	spinal muscular atrophy
SMA-PME	spinal muscular atrophy with progressive myoclonic epilepsy
SMPD1	lysosomal sphingomyelin phosphodiesterase 1
SMPD2	neutral sphingomyelin phosphodiesterase 2
Sph	sphingosine
Sph-1P	sphingosine-1-phospate
