# Supplementary material for: Acid Ceramidase Depletion Impairs Neuronal Survival and Induces Morphological Defects in Neurites Associated with Altered Gene Transcription and Sphingolipid Content
Source: Int J Mol Sci. 2020 Feb 26;21(5):1607. doi: 10.3390/ijms21051607 (PMC7084529; doi:10.3390/ijms21051607)
Supplement: Supplementary file 1 [file ijms-21-01607-s001.pdf]

International Journal of
Molecular Sciences

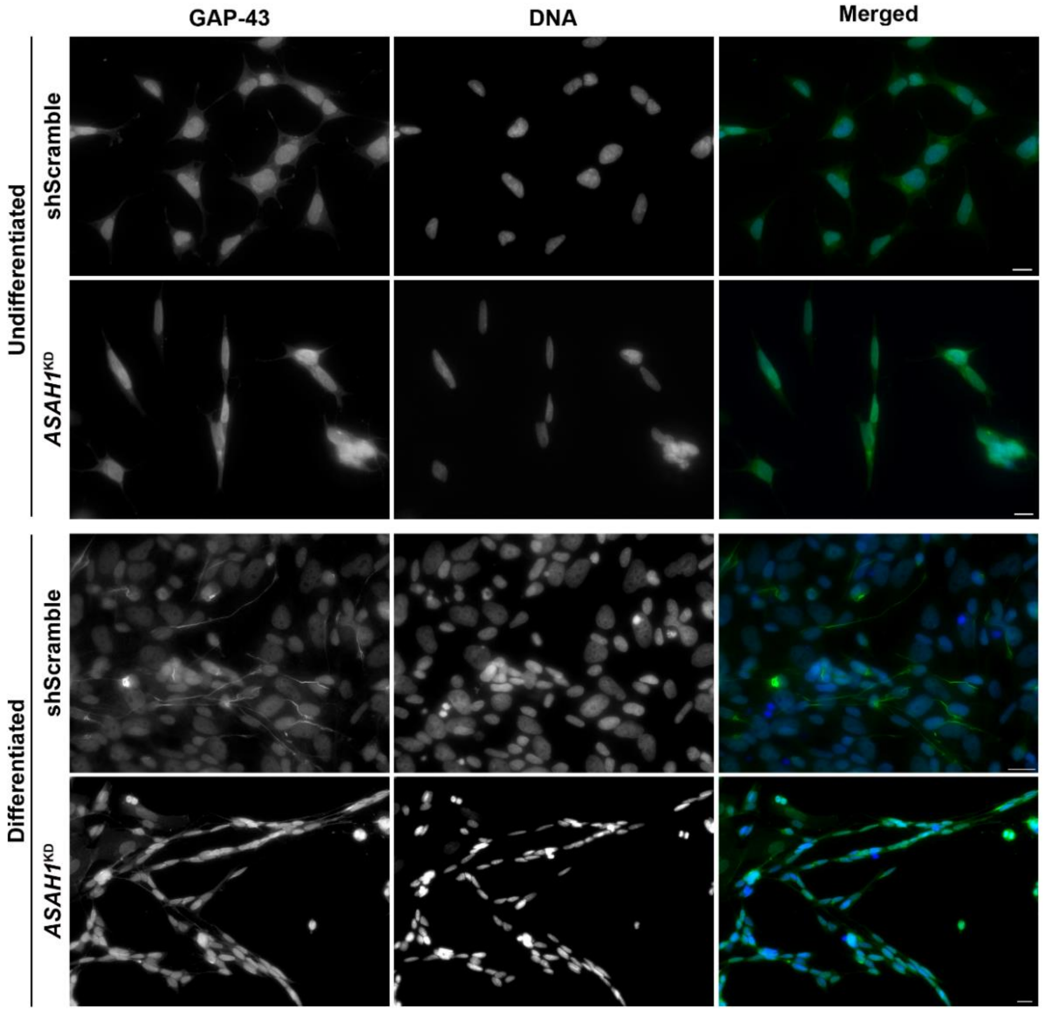

Figure S1. Both shScramble and *ASAH1*^{KD} cells display the ability to differentiate after the treatment with all-*trans* retinoic acid. Immunofluorescence microscopy images of undifferentiated and differentiated shScramble and *ASAH1*^{KD} cells stained with the GAP-43 marker (green in merged image). Nuclei were stained with Hoechst dye (blue in merged image). The scale bars correspond to 20 μm.

Table S1. Primers that were used for reverse-transcription quantitative PCR.

Oligonucleotide sequence (5′ - 3′)	F/R*	Target gene
CGTTGGCTGCTAGAGCGATG	F	*ASAH1*
CTGCACCTCTGTACGTTGGTC	R	*ASAH1*
GCATCAACACAGGAGAGTC	F	*ASAH2*
GGAGGCAGAGGCATAGAG	R	*ASAH2*
CTTTCGGAATGACCGGGGTA	F	*ACER2*
GCATACACAGCCCAGGTAGG	R	*ACER2*
CAATGTTCGGTGCAGTTCAGAG	F	*ACER3*
GGATCCCATTCCTACCACTGTG	R	*ACER3*
AGTCCACCACAACAGCAC	F	*CERK*
GAGGAAGGTCTTTAAACCTG	R	*CERK*
TGGTTCCTGTACATCGTGGC	F	*CERS1*
CTCGGCTGTGTCATACTCCC	R	*CERS1*
CATCGTCTTCGCCATTGTTT	F	*CERS2*
TTGTTATTGAGGATGGGGTG	R	*CERS2*
GCTCTTCGAGCGATTTATTGCC	F	*CERS5*
GGCCCTCCAGCCTTTTCTTA	R	*CERS5*
GCTGACGAGGTTCTGTGAG	F	*CERS6*
AGTTGTGAGTGGCTGATAGG	R	*CERS6*
TGAGATTGCCCACAATGCTGC	F	*DEGS1*
TACATCGACGCCATCAGCTCCAAG	R	*DEGS1*
GCCAGGACTTGATCAACCTAACC	F	*SGMS1*
CCATTGGCATGGCCGTTCTTG	R	*SGMS1*
CTGACTCTCGGGTTCTCTGG	F	*SMPD1*
AGGTTGATGGCGGTGAATAG	R	*SMPD1*
CTGGGGCATTCCGTACTTGA	F	*SMPD2*
CTGGAAGTCCTGCTCACTCC	R	*SMPD2*
GAA GGT GAA GGT CGG AGTC	F	*GAPDH*
GAA GAT GGT GAT GGG ATTTC	R	*GAPDH*
ACCCTAGGGGAGACACACCG	F	*GBA1*
AATTGGGTCCTCCTTCGGGG	R	*GBA1*
TGGGCGTCTCTAATGTCTGC	F	*LAMP1*
CAGGATCACCCCGAATGTCA	R	*LAMP1*
ATCGCCTGAGGCCCCTCTCC	F	*RHOA*
GCTCCCGCCTTGTGTGCTCA	R	*RHOA*
AAC CTCCCG GGGCAAAGACAAG	F	*RAC1*
AGTGTTGGGACAGTGGTGCCG	R	*RAC1*
ATGTTTTTGCAAGCAGTCAAGGA	F	*DIAPH1*
ATCACACCTGTCTCATCGCC	R	*DIAPH1*
CACCACTGTCCAAAGACTCCT	F	*CDC42*
CTGCGGCTCTTCTTCGGTTC	R	*CDC42*

*F/R: Forward/Reverse.

Table S2. Primary and secondary antibodies that were used in immunofluorescence and Western blot experiments.

	General information					Dilution	
	Name	M/P[a]	Source	Supplier Details	Catalog Number	I.F[b]	W.B[c]
Primary	ASAH1	P	Rabbit	Sigma	HPA005468	-	1:1000
	Bax (2D2)	M	Mouse	Santa Cruz	Sc-20067	-	1:200
	Bcl-2 (C-2)	M	Mouse	Santa Cruz	Sc-7382	-	1:200
	Cyclin D1	M	Mouse	Abcam	ab6152	-	1:300

	GAP43 (7B10)	M	Mouse	Santa Cruz	Sc-33705	-	1:100
	GAPDH (6C5)	M	Mouse	Santa Cruz	Sc-32233	-	1:1000
	LAMPI (D2D11)	M	Rabbit	Cell Signaling Technology	9091P	1:500	1:2000
	TUB-b (E7-c)	M	Mouse	Hybridoma Bank	P07437	1:200	-
	β-Actin(C4)	M	Mouse	Santa Cruz	Sc-47448	-	1:500
Secondary	CF™488A	P	Mouse IgG	Biotium	20014	1:500	-
	CF™568	P	Rabbit IgG	Biotium	20339	1:500	-
	Peroxidase conjugated	P	Mouse IgG	Jackson Immunoresearch	115-035-003	-	1:2000
	Peroxidase conjugated	P	Rabbit IgG	Jackson Immunoresearch	111-035-003	-	1:2000

[a]M/P: Monoclonal/Polyclonal, [b]I.F: Immunofluorescence, [c]W.B: Western Blot.

Table S3. Summary of two-way ANOVA analysis details for all experiments.

**Figure**	**F value**	**p value for interaction**	**Post-hoc test**	**p value for multiple comparisons (ASAH1^{KD} compared to shScramble**
**2**	3.968	0.0320	Dunnett's multiple comparisons test	**At 24h:** 0.4982 for sh*ASAH1-1* and 0.9933 for sh*ASAH1-2*
				**At 48h:** 0.1323 for sh*ASAH1-1* and 0.9794 for sh*ASAH1-2*
				**At 72h:** 0.0039 for sh*ASAH1-1* and >0.9999 for sh*ASAH1-2*
**3**	30.65	<0.0001	Sidak's multiple comparisons test	0.9949 for debris
				<0.0001 for G1 phase
				0.0257 for S phase
				<0.0001 for G2 phase
				0.2710 for aggregates
**8**	16.41	0.0009	Sidak's multiple comparisons test	0.0026 for *PHOA*
				0.1321 for *RAC1*
				0.0702 for *CDC42*
				0.1304 for *DIAPH1*
**9A**	2.965	0.0126	Sidak's multiple comparisons test	>0.9999 for C14-Cer
				0.9114 for C16-Cer
				0.8887 for C18-Cer
				0.9963 for C18:1-Cer
				>0.9999 for C20-Cer
				>0.9999 for C20:1-Cer
				0.9994 for C22-Cer
				>0.9999 for C22:1-Cer
				>0.9999 for C24-Cer
				<0.0001 for C24:1-Cer
				>0.9999 for C26-Cer
				>0.9999 for C26:1-Cer
**9B**	31.95	<0.0001	Sidak's multiple comparisons test	>0.9999 for dhC14-Cer
				>0.9999 for dhC16-Cer
				>0.9999 for dhC18-Cer

				>0.9999 for dhC18:1-Cer
				<0.0001 for dhC20-Cer
				0.9974 for dhC20:1-Cer
				>0.9999 for dhC22-Cer
				0.9999 for dhC22:1-Cer
				>0.9999 for dhC24-Cer
				0.9913 for dhC24:1-Cer
				>0.9999 for dhC26-Cer
				>0.9999 for dhC26:1-Cer
**9C**	1.75	0.1297	Sidak's multiple comparisons test	>0.9999 for C14-HexCer
				0.0107 for C16-HexCer
				>0.9999 for C18:1-HexCer
				>0.9999 for C18-HexCer
				>0.9999 for C20:1-HexCer
				>0.9999 for C20-HexCer
				>0.9999 for C22:1-HexCer
				>0.9999 for C22-HexCer
				0.1464 for C24:1-HexCer
				0.6144 for C24-HexCer
				>0.9999 for C26:1-HexCer
**9D**	11.94	<0.0001	Sidak's multiple comparisons test	0.9997 for C14-SM
				<0.0001 for C16-SM
				>0.9999 for C18-SM
				>0.9999 for C18:1-SM
				>0.9999 for C20-SM
				>0.9999 for C20:1-SM
				>0.9999 for C22-SM
				>0.9999 for C22:1-SM
				>0.9999 for C24-SM
				0.8565 for C24:1-SM
				>0.9999 for C26-SM
				>0.9999 for C26:1-SM
**9E**	0.6607	0.76	N/A	N/A
**9F**	16.76	0.0008	Sidak's multiple comparisons test	0.9961 for dhSph
				>0.9999 for dhSph-1P
				0.0001 for Sph
				>0.9999 for Sph-1P
**10**	16.29	<0.0001	Sidak's multiple comparisons test	<0.0001 for *CERK*
				0.1254 for *SMPD1*
				0.3192 for *SMPD2*
				0.0484 for *SGMS1*
				<0.0001 for *DEGS1*
				0.0092 for *GBA*
				0.1178 for *ASAH2*
				0.2611 for *ACER2*
				0.1554 for *ACER3*
				0.0008 for *CERS1*
				<0.0001 for *CERS2*
				0.5859 for *CERS5*

	0.3709 for *CERS6*
